# Diagnostic yield of early versus late outpatient Cardiac MRI in suspected acute coronary syndrome in patients with normal coronary arteries

**DOI:** 10.1186/1532-429X-15-S1-E67

**Published:** 2013-01-30

**Authors:** Daniel Swarbrick, Rajiv Das, Nicholas M Child

**Affiliations:** 1 Cardiothoracic, Freeman Hospital, Newcastle upon Tyne, UK

## Background

Cardiac MRI (CMR) is increasingly recognized as a valuable imaging modality for the investigation of patients with suspected acute coronary syndrome found to have non obstructive coronary arteries on angiography. Studies have shown that early CMR within 48 hours can facilitate diagnosis of important differential diagnoses, particularly myocarditis. We attempted to demonstrate whether later scanning, after 6 weeks, would still have a similar pick up rate for these diagnoses, compared with earlier CMR within 6 weeks.

## Methods

64 patients with suspected acute coronary syndrome referred for coronary angiography at a tertiary cardiac centre who had normal coronary arteries enrolled to the study and were entered into a prospective registry. All underwent cardiac MRI. Axial black blood, multiplanar cines, STIR and delayed enhancement sequences were taken. The first 32 patients had cardiac MRI performed after 6 weeks, and the subsequent 32 had MRI performed within 6 weeks. The two groups were compared with respect to diagnoses made on MRI, as well as demographics and baseline troponin concentrations.

## Results

32 patients underwent CMR scanning after 6 weeks, at an average of 50.5 days. The final diagnoses were myocardial infarction (36%), myocarditis (13%), Takotsubo cardiomyopathy (6%) and non-diagnostic (45%). The subsequent 32 patients underwent CMR within 6 weeks, at an average of 22.6 days. The final diagnoses in this group were myocardial infarction (19%), myocarditis (41%), Takotsubo cardiomyopathy (13%), non-diagnostic (25%) and hypertrophic cardiomyopathy (single case).

## Conclusions

Outpatient CMR performed on patients with suspected acute coronary syndrome but with normal coronary arteries later than 6 weeks has a lower diagnostic yield compared to early outpatient CMR within 6 weeks. The pick up rate for myocarditis on CMR within 6 weeks is comparable to that in previous studies of CMR performed within 48 hours of admission, suggesting that it is feasible to perform scans during this time without compromising diagnostic yield.

## Funding

No external funding was received.

